# ZnSe nanotrenches: formation mechanism and its role as a 1D template

**DOI:** 10.1186/1556-276X-6-272

**Published:** 2011-03-30

**Authors:** Gan Wang, Shu Kin Lok, Iam Keong Sou

**Affiliations:** 1 Nano Science and Technology Program, The Hong Kong University of Science and Technology, Clear Water Bay, Kowloon, Hong Kong, China; 2 Department of Physics, The Hong Kong University of Science and Technology, Clear Water Bay, Kowloon, Hong Kong, China

## Abstract

High-resolution transmission electron microscopy was used to characterize the microstructures of ZnSe nanotrenches induced by mobile Au-alloy droplets. The contact side interfaces between the AuZn_δ _alloy droplets and the ZnSe as well as the four side walls of the resulting <011>-oriented nanotrenches were found all belong to the {111} plane family, with the front and back walls being the {111}A planes while the other two side walls being the {111}B planes. These findings offer a deeper understanding on the formation mechanism of the nanotrenches. Pure Au nanodashes were formed upon further deposition of Au on the nanotrenches.

**PACS**: 61.46.Df, Structure of nanocrystals and nanoparticles. 81.16.Rf, Micro and nanoscale pattern formation. 68.37.Og, High resolution transmission electron microscopy.

## Introduction

As length scales decrease below the range easily accessible by lithographic patterning, there is great interest in developing processes to form surface structures spontaneously [1]. Among the different approaches used for fabricating nanostructures, deposition of functionalized materials into patterned nanotrenches on a substrate has attracted increasing interest. This approach has been applied to various applications, such as chemical sensing, dimensional crossover influence in granular electronic systems, heterojunction tunneling field effect transistors, and precise quantum dot placement [2-6]. Fabrication of nanotrenches structures can be achieved by a number of different ways, such as electron-beam lithography [7], focused ion beam [2,8] milling, and nanoimprint lithography [5,9]. These three approaches enjoy the advantage of being able to create highly ordered patterns; however, they suffer from the need of much time-consuming and contaminating processing. Using metal-assisted-chemical-reaction etching without fluoride, Sun and Akinaga [10] have fabricated noodle-like nanotrenches on porous silicon substrates. However, they were not highly aligned and ordered, and it was difficult to reach a truly nanoscale width. Byon and Choi [11] have demonstrated using single-walled carbon nanotubes (SWNTs) to selectively etch one-dimensional nanotrenches in SiO_2_. The shape, length, and trajectory of the nanotrenches are fully guided by the SWNTs. The challenge for realizing ordered nanotrenches using this approach will be the need for sophisticated techniques that permit the alignment of the carbon nanotubes. Recently, some mobile metallic nanoparticles (NPs) were found to act as catalyst to induce nanotrench formation. Byon and Choi [12] reported that Fe NPs could initiate the carbothermal reduction to form SiO_2 _nanotrenches. In the recent years, using the state of the art molecular beam epitaxy (MBE) technique, we have been able to study the growth mechanism and the quantum size effects of several self-assembled nanostructures [13-15]. Recently, we reported that highly aligned nanotrenches were produced during the thermally agitated migration of AuZn_δ _alloy droplets through a catalytic reaction with an underlying ZnSe thin film [16]. More recently, Amalric et al. [17] further reported that nucleation of Au catalyst in ZnSe nanotrenches assists the growth of ZnSe and ZnSe/CdSe nanowires preferentially in directions orthogonal to the trenches. In this study, we report high-resolution transmission electron microscopy (HRTEM) imaging of Au-alloy droplet-driven ZnSe nanotrenches, which provides a deeper understanding on the nanotrench formation mechanism. The use of the nanotrenches as a template for fabricating Au nanodashes is also presented.

## Experiment

In this study, the samples were fabricated on GaAs (100) substrates in a VG V80H MBE system. A ZnSe layer (100 nm) was first grown at 250°C using a ZnSe compound source. Sample #1 was then deposited with a 0.45-nm Au at 150°C followed by a thermal annealing at 550°C for 20 min to generate the nanotrenches. Sample #2 was deposited with a 0.23-nm Au layer instead so as to generate narrower nanotrenches. After the thermal annealing, sample #2 was then cooled down to 500°C followed by a further Au deposition of 0.9 nm with the expectation of forming Au nanostructures within the nanotrenches. A JEOL 2010F HRTEM and a JEOL JSM6700F high-resolution scanning electron microscope (HRSEM) were used for structural characterization. Chemical analysis was performed using the energy-dispersive X-ray spectroscopy (EDS) facility built into the HRTEM.

## Results and discussion

In a recent article, Amalric et al. [17] reported that some short trenches with irregular shape mainly oriented along the <011> direction were observed to present at a bare ZnSe surface at temperature ≥400°C. They argued that the trenches are more probably related to a sublimation mechanism of the ZnSe layer alone. However, they also observed that with the presence of Au NPs at the ZnSe surface, annealing at 530°C can generate much longer and well-aligned trenches with the AuZn_δ _particles all localized at the extremities of the trenches the same as what we have reported earlier [16]. In a recent report on the <011>-oriented self-assembled formation of nano-groove structure at the surface of an annealed Fe/ZnSe bilayer [18], we have also pointed out that a bare ZnSe surface annealed at high temperature can itself generate an imperfect nano-groove structure; however, the presence of the Fe catalyst layer plays a role in enhancing the formation of the 1D nanostructure to a great extent in its perfection at a lower annealing temperature. We believe that the above observations are all correlated with each other confirming that annealing of a bare ZnSe surface can induce an imperfect <011>-oriented trench/groove structure to a certain extent being attributed to the minimization of the surface energy. The migration of the AuZn_δ _NPs and their induced catalytic decomposition of the several top layers of ZnSe lead to the formation of the long and well-aligned nanotrenches, similar to the role of the Fe catalytic layer in enhancing the formation of the nano-groove structure. In our most recent top-view SEM study, it was found that if a bare ZnSe surface was heated to a certain high temperature, then some Se dots with perfectly spherical shape were generated. Figure S1 in Additional file 1 shows the SEM image of these Se dots. With the presence of AuZn_δ _NPs, the induced nanotrenches were found to penetrate across the Se dots that were observed to be distorted into an elongated shape being attributed to the cross-over migration of the AuZn_δ _NPs. Figure S2 in Additional file 2 shows the SEM image of the distorted Se dots resting on the nanotrenches passing through them. This provides further evidence that the long and well-aligned nanotrenches were indeed induced by the migration of the NPs and their catalytic decomposition of ZnSe.

**Figure 1:**
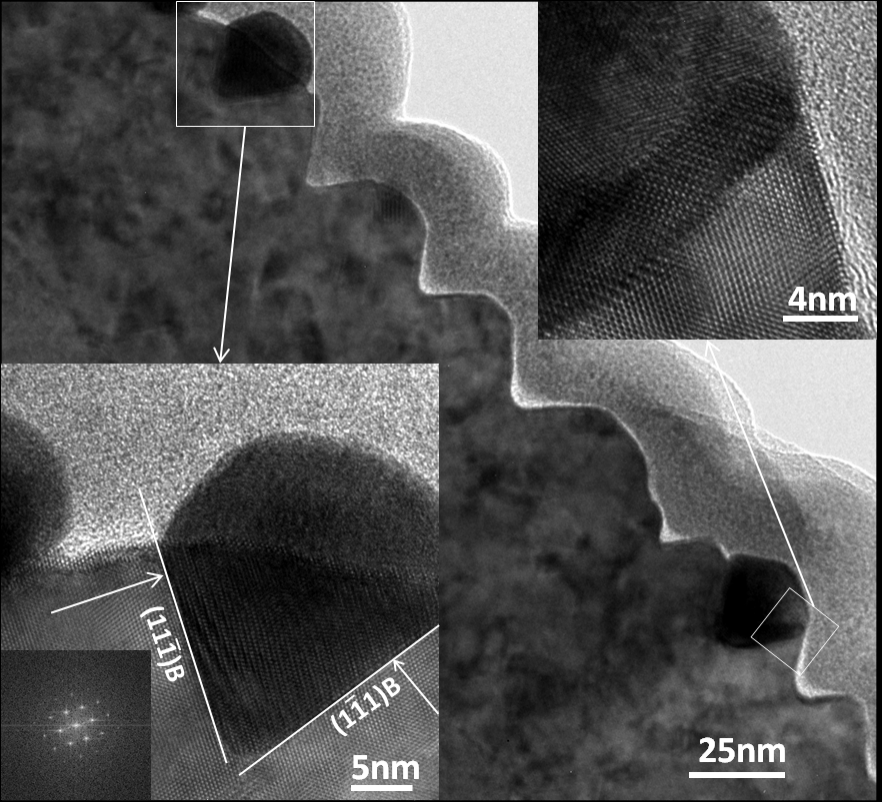
**Cross-sectional TEM image of nanotrenches with the viewing zone axis along [011] orientation**. Bottom-left inset shows the HRTEM image taken for the AuZn_δ _NP on the left side of this figure with a Fourier transform pattern of the nearby ZnSe lattice. Top-right inset shows the HRTEM image of the AuZn_δ _NP on the right side of this figure.

Figure 1 shows a cross-sectional TEM view of a number of nanotrenches on a piece cut from sample #1 with the viewing zone axis along the [011] direction, that is, along the nanotrench orientation; we term this as a front view observation. The AuZn_δ _NPs of two of these nanotrenches are by chance located in the viewing zone of this cross-sectional sample, while the rest of them just display the front view of the "empty" trench body. One can see that the front view cross section of the nanotrenches has a V shape in general, while that of the AuZn_δ _NPs has a V shape for the portion embedded in the ZnSe layer and an arc shape for the portion above the trench body. The bottom-left inset in Figure 1 shows an HRTEM image of the AuZn_δ _NP on the left side of this figure. In this inset, a Fourier transform pattern of the ZnSe lattice near the NP is also shown. Using the Fourier transform pattern as references, both the interfaces of the V shape are found to be the members of the {111} plane family of ZnSe as indicated in the bottom-left inset of Figure 1. In a previously published article, we have identified that the nanotrenches are along either the [011] or  directions that are anti-parallel with each other, in which the identification was based on the orientation of the resulting nanotrenches formed on a GaAs(100) substrate with a pretilting angle of 2° off toward the [111]A direction [16]. Figure S3 in Additional file 3 shows the planar representation of the orientation relationship of the crystal planes of the ZnSe(100) layer, which is deduced from the relevant data given by the manufacturer of the GaAs(100) wafers used in this study. As can be seen in Figure S3, the interfacial planes of the V shape shown in Figure 1 are B plane and B planes, respectively, and both are Se-terminated planes. The top-right inset in Figure 1 shows the HRTEM image of a portion of the AuZn_δ _NP on the right side of this figure. The moire fringes located near the V-shaped region within the NP together with the regular lattice pattern in the rest of the NP region indicate that it is single crystalline. We have performed separately a detailed analysis on the microstructure of a few NPs of this sample using the built-in electron diffraction technique. It was found that the NPs are FCC structures with various orientation relationships with the underlying ZnSe lattice and their lattice constants are slightly smaller than that of pure Au lattice being attributed to the inclusion of small amount of Zn as reported in our previous publication [16].

**Figure 2:**
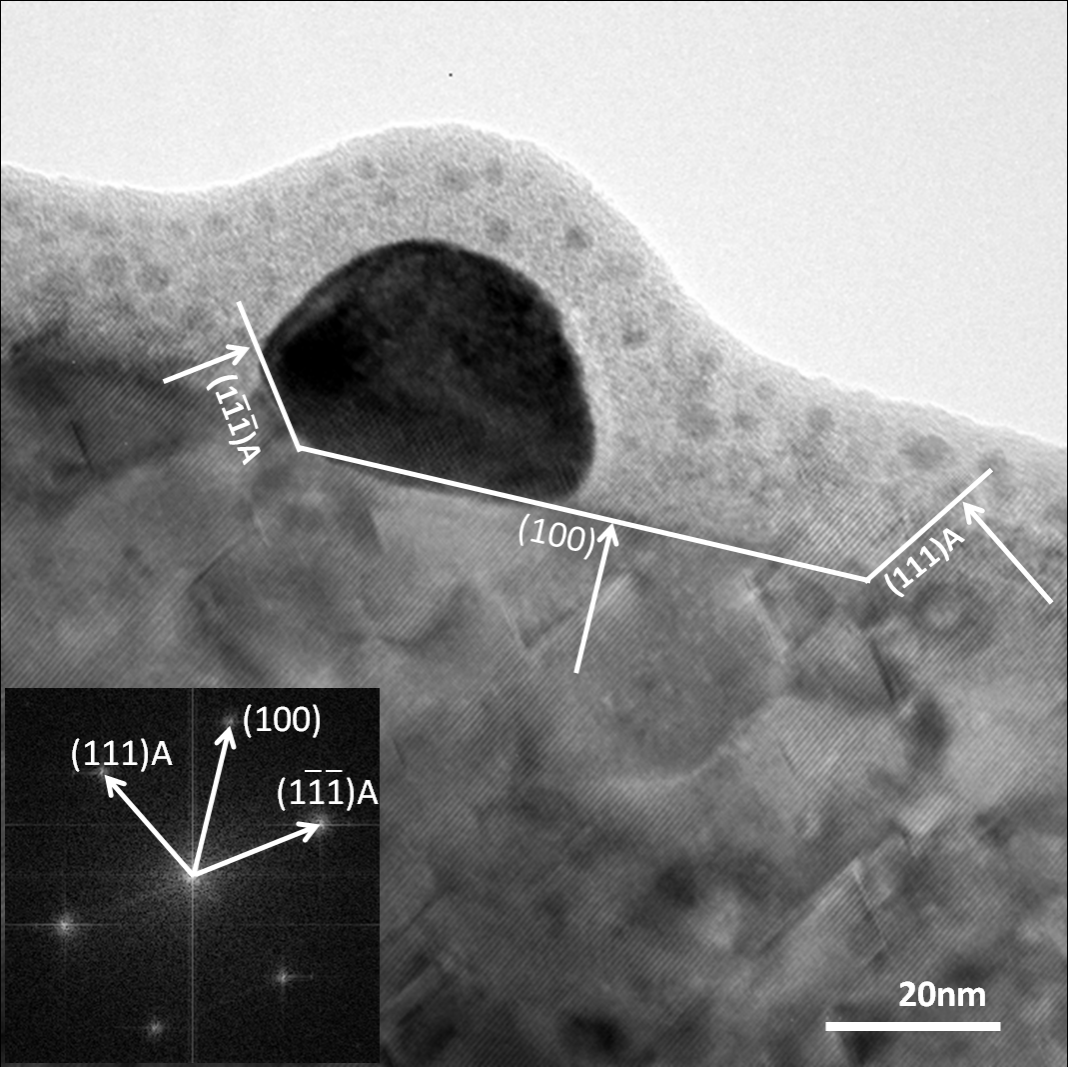
**Cross-sectional TEM image of a nanotrench with the viewing zone axis 90° off [011] orientation**. Inset shows the Fourier transform pattern taken from the nearby ZnSe lattice.

The side-view cross-sectional HRTEM image of a nanotrench with the viewing zone at 90° off the [011] direction, that is, perpendicular to the nanotrench orientation, is shown in Figure 2. This side-view image together with the Fourier transform pattern of the ZnSe lattice as shown in its inset reveals that the left contact interface between the NP and the ZnSe lattice and the right-end surface of the nanotrench are both members of Zn-terminated {111}A surface family. From Figure S3 in Additional file 3 they can be determined to be either the (111)A or the A plane. It is also worthy to note that the non-contacted portion of the surface of the NP is of an arc shape as can be seen in Figure 2.

The HRTEM observations described above offer more insightful details than what we have reported previously on the formation mechanism of the nanotrenches induced by the mobile catalytic particles. Our further understanding on the formation mechanism is illustrated as follows. At the annealing temperature, Au droplets first react with the ZnSe thin film to form AuZn_δ _alloy droplets. During this process, the droplets fall into the ZnSe layer by a fraction of their size. As described earlier, the portion fell into the ZnSe lattice has four contact surfaces, all of them belong to the {111} plane family. In our previously published article regarding the study on the growth mechanism of ultra-thin ZnSe nanowires using Au NPs as the catalyst, we have shown that the interfaces between the catalyst particles and the ZnSe NWs were always {111} planes regardless of whether their growth directions are along [111], [211], or [110]. We have argued that this feature is likely driven by the minimization of the total energy of the nanowire system and the fact that {111} planes of ZnSe have the lowest interface energy [15]. We believe that all the four contact surfaces of the AuZn_δ _catalyst droplets for the formation of the nanotrenches represent {111} planes because of the same origin of driving force as just described for the growth of ZnSe nanowires. The observed arc shape of the non-contacted portion of the AuZn_δ _catalyst droplets shares the same cause as well since it is well known that a spherical shape for a non-contacted nanodroplet has the smallest surface area so as to minimize its surface energy.

**Figure 3:**
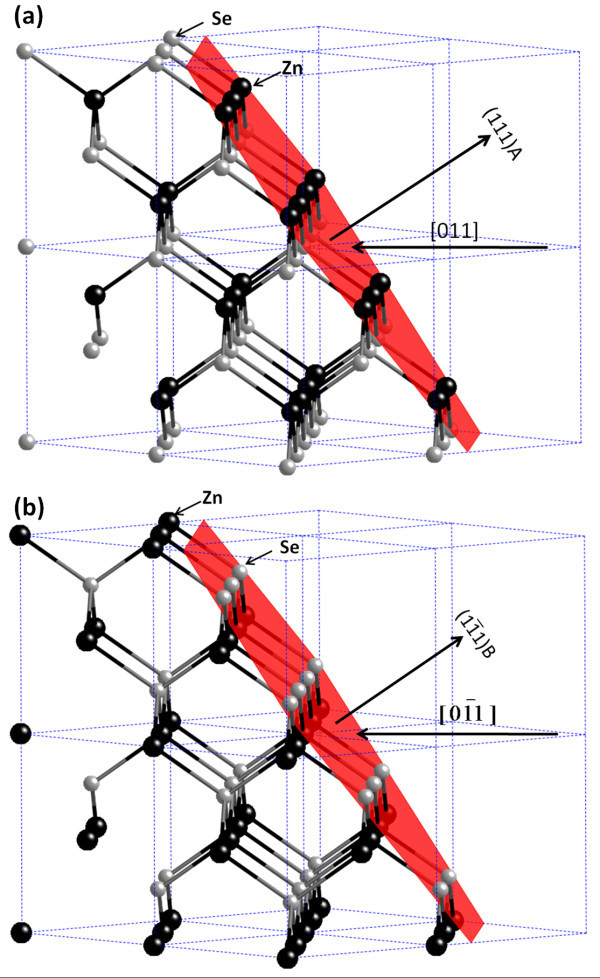
**Tilted-view schematic diagrams of ZnSe lattice: **(a) **along [011] and **(b) **along  direction**.

In our previously published article, we have discussed the reason for the nanotrenches induced by the migration of AuZn_δ _being only oriented along a specific pair of <011> direction although there are four <011> directions on the surface of a (100)-oriented substrate of zinc-blended structure [16]. This is because the [011]/ and the  pairs are not identical because of the inversion symmetry on the (100) plane of a zinc-blended structure. As viewed along the [011] and  directions, the zigzag atomic chains presented on the viewing planes are in fact 180° off with regard to the location of the Zn and Se atoms, with Zn atoms at the top as viewed along the [011] direction while Se atoms at the top as viewed along the  direction. We further argue that AuZn_δ _droplets prefer to attack Zn atoms more than Se atoms because it is more energetically favorable because the heat of formation of Au-Zn (-0.27 eV/atom) [19] is lower than that of Au-Se (-0.15 eV/atom) [20]. This study further reveals that the contact interfaces between the AuZn_δ _droplet and the ZnSe lattice are {111}A and {111}B planes for the [011]/ and the  pairs, respectively, which in fact provides further evidence in support of our explanation described above. Figure 3a, b displays the tilted views of a ZnSe lattice as viewed along the [011] and  directions, with the top surface terminated at (111)A and  B, respectively. These schematic drawings are applicable to the views along the  and  directions as well. The inclined top surfaces represent the direct contact surface between a AuZn_δ _droplet and the ZnSe lattice. As can be seen in Figure 3, the contact surfaces for the [011]/ directions are Zinc terminated, while those for the  directions are Se terminated. Being attributed to the difference between the heat of formation of Au-Zn and Au-Se, the [011]/ directions represent the preferred directions for the formation of the ZnSe nanotrenches since the migration of the AuZn_δ _droplets and their catalytic decomposition reaction are more favorable along these anti-parallel directions than along the  directions.

**Figure 4:**
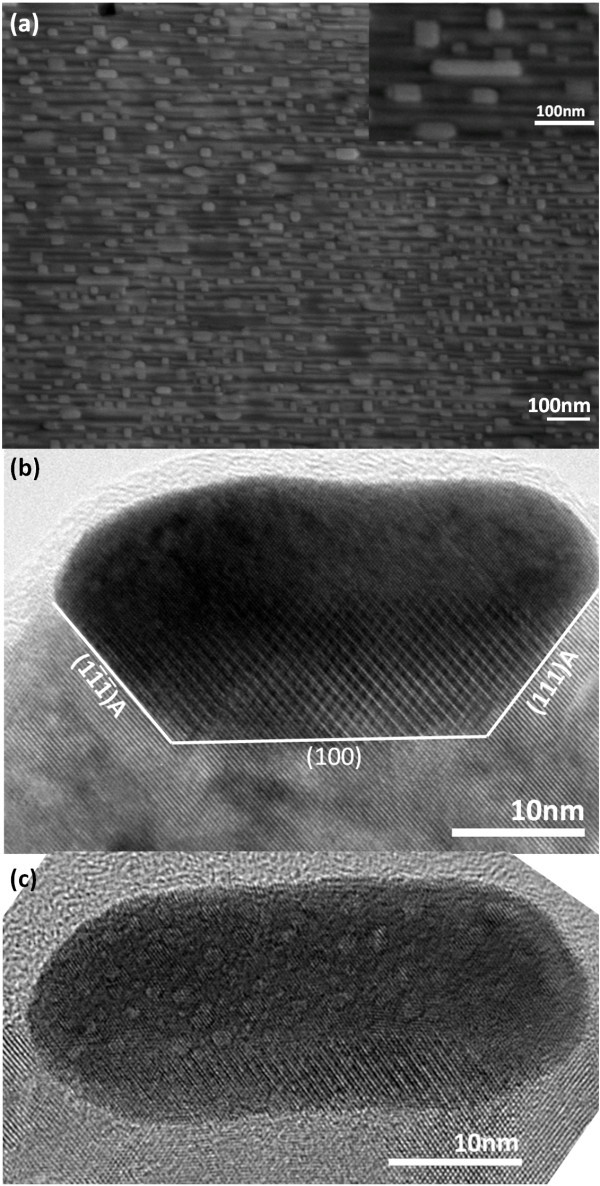
**Electron microscopic images of Au nanostructures being filled into the nanotrenches: (a)** The plan-view SEM image. Inset displays one of the Au nanodashes of 140 nm in length; **(b) **the cross-sectional TEM image taken from a nanodash that has completely filled up the underlying nanotrench; **(c) **a nanodash located within a nanotrench with both the front and back surfaces being non-contacted. The viewing zone axis of **(b, c) **is perpendicular to the nanotrenches.

Recently, Xue et al. [21] have demonstrated the fabrication of ultrafine protein arrays on Au nanowires arrays through the interactions of protein-mercaptoundecanoic acid and gold. In this study, using a sample with aligned nanotrenches as a template, further Au deposition of 9.1 Ǻ in nominal thickness was carried out at a lower growth temperature with the expectation that the deposited Au in the second growth step may fall into the nanotrenches to form 1D Au nanostructure. Figure 4a shows the SEM image of a typical resulting surface of this sample, which is named as sample #2. One can see that the resulting nanotrenches are partially filled with high-density nanostructures of which their top-view shapes are either square or rectangle with sharp corners, which are in high contrast with the spherical shape of the catalyst particles. Some of these nanostructures have higher aspect ratio, although they are rare. The inset in Figure 4a shows one of these "nanodashes" with a length of about 140 nm. Figure 4b displays the HRTEM images of a completely filled-in nanodash with both the front and back contact surfaces being the {111}A planes while Figure 4c displays one that is located within a nanotrench with both the front and back surfaces being non-contacted with arc shapes. The shapes of the contact surfaces and the non-contacted surfaces of the filled-in nanostructures shown in these images offer further evidence that the shape of the filled-in nanostructures is also driven by the minimization of the system energy. One thing is worth pointing out that both subsequent EDS analysis and a detailed study performed on the Fourier transform pattern taken at the regular lattice pattern of the nanodash shown in Figure 4c reveal that the filled in material is pure Au with epitaxial relationship of [100]_Au_//[100]_ZnSe _in contrast to the AuZn_δ _alloy phase and the lattice misalignment of the catalytic droplets. It is believed that the nanodashes filled in the nanotrenches are pure Au instead of AuZn_δ _alloy because a lower substrate temperature of 500°C was used for the secondary Au deposition that only lasts for 2.5 min, which lacks sufficient energy to initiate the Au-Zn alloying process, whereas the first Au deposition having been annealed at 550°C for 20 min is capable of resulting in the formation of AuZn_δ _alloy NPs. The formation of Au nanodashes demonstrated in this study indicates that it is indeed possible for using the ZnSe nanotrenches as a template to fill in other materials to form novel low-dimensional nanostructures.

## Conclusions

In summary, the three-dimensional shapes of ZnSe nanotrenches induced by mobile AuZn_δ _droplets were investigated using cross-sectional HRTEM imaging technique, revealing that the contact side interfaces between the AuZn_δ _alloy droplets and the ZnSe lattice are all belong to the {111} plane family. The front and back walls of the resulting <011>-oriented nanotrenches were found to be Zn-terminated {111}A planes while the other two side walls are Se-terminated {111}B planes. These findings further provide the explanation for the [011]/ directions being the preferred directions for the formation of the ZnSe nanotrenches. We have also demonstrated the formation of pure Au nanodashes inside the nanotrenches. Further study is being carried out in our laboratory to investigate the possibility of forming 1D nanostructures of other materials using the developed nanotrenches as a highly aligned template.

## Abbreviations

EDS: energy-dispersive X-ray spectroscopy; HRSEM: high-resolution scanning electron microscope; HRTEM: high-resolution transmission electron microscopy; MBE: molecular beam epitaxy; NPs: nanoparticles; SWNTs: single-walled carbon nanotubes.

## Competing interests

The authors declare that they have no competing interests.

## Authors' contributions

GW participated in the design of the study, MBE growth, HRSEM, and HRTEM analysis and drafted the manuscript. SKL participated extensively in HRTEM imaging and experimental data analyses. IKS coordinated the design of the study, proposed the phenomenological model and significantly contributed to the drafting of this manuscript. All the authors have read and approved the final manuscript.

## Supplementary Material

Additional file 1 **Figure S1**. SEM image of the round dots resulted from a bare ZnSe surface annealed at 550°C for 10 min. Separate EDS analysis performed on these dots reveals that they are Se dots. Click here for file

Additional file 2 **Figure S2**. SEM image of the distorted Se dots passed through by nanotrenches. The inset is an AFM image that reveals the dark spots in this SEM image are indeed elongated particles. Click here for file

Additional file 3 **Figure S3**. Planar representation of the orientation relationship of the crystal planes of the ZnSe(100) layer. Click here for file
