# Supplementary figures and images for: ZnSe nanotrenches: formation mechanism and its role as a 1D template
Source: Nanoscale Res Lett. 2011 Mar 30;6(1):272. doi: 10.1186/1556-276X-6-272 (PMC3211336; doi:10.1186/1556-276X-6-272)

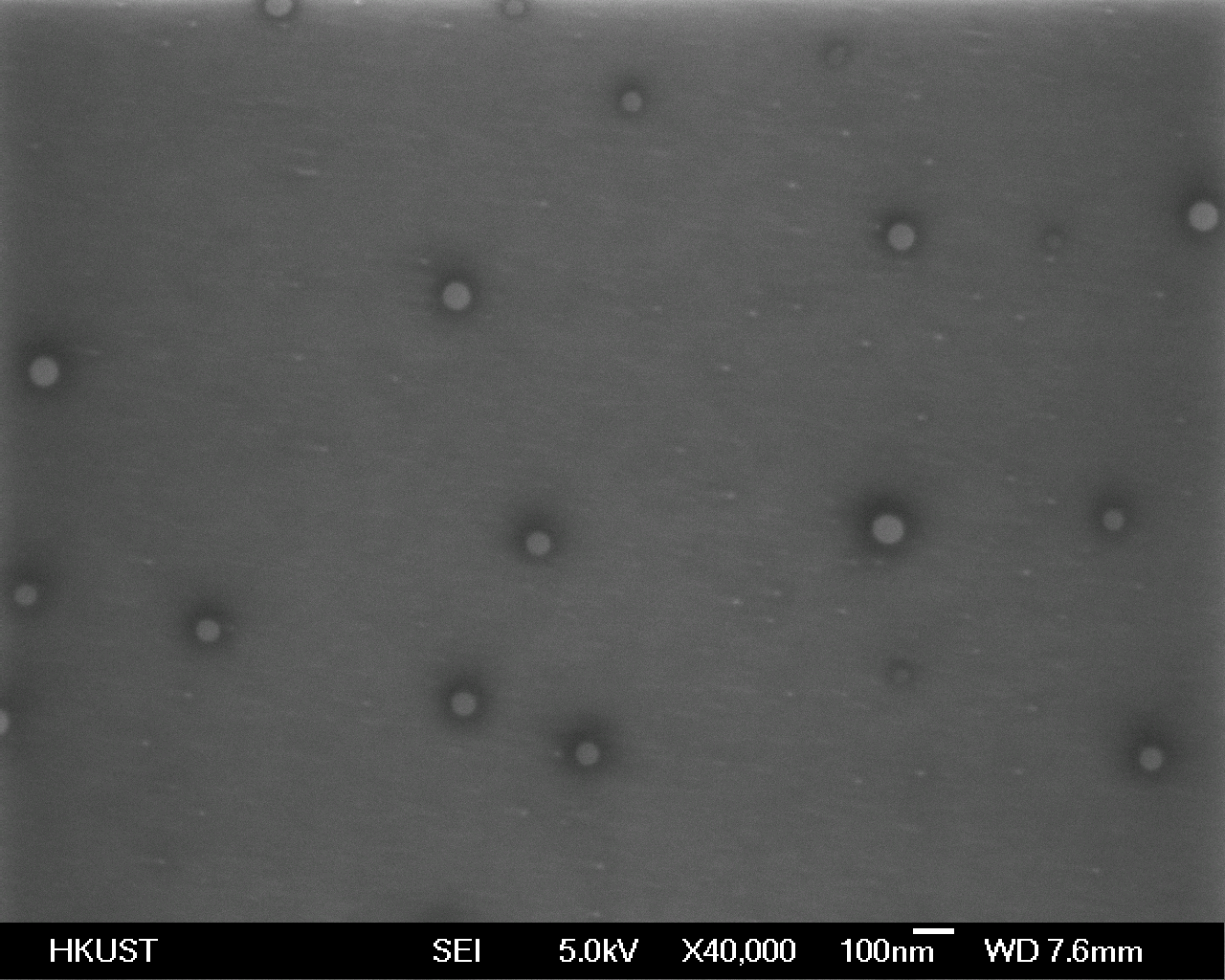

Supplement: Additional file 1 — Figure S1. SEM image of the round dots resulted from a bare ZnSe surface annealed at 550°C for 10 min. Separate EDS analysis performed on these dots reveals that they are Se dots. [file 1556-276X-6-272-S1.TIFF]

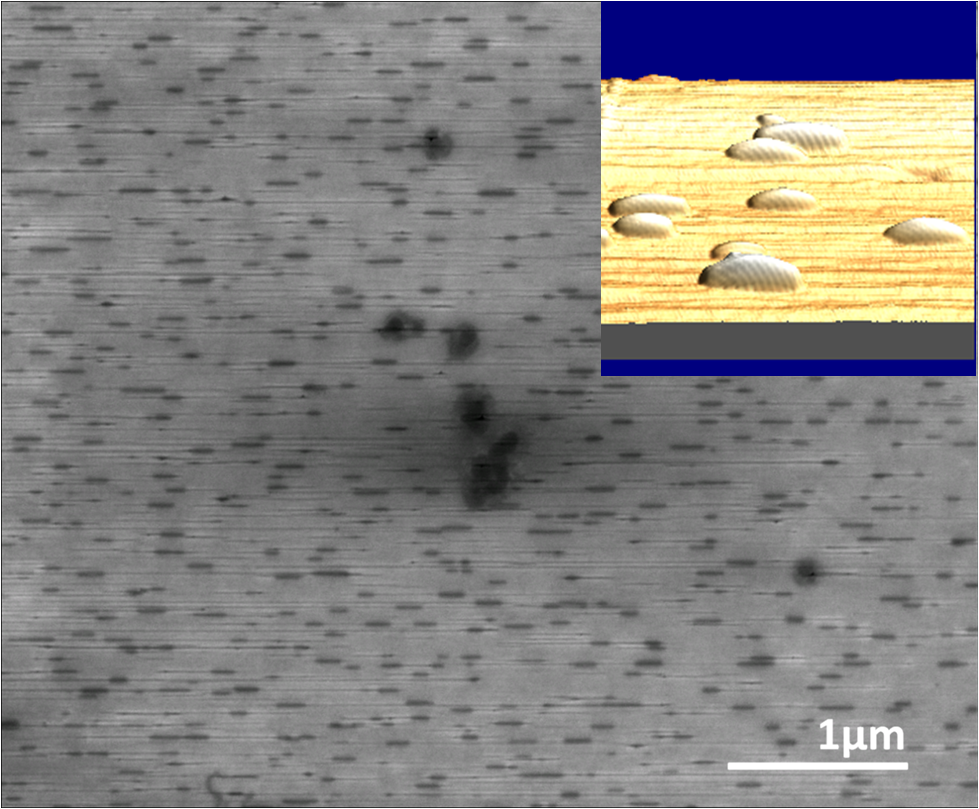

Supplement: Additional file 2 — Figure S2. SEM image of the distorted Se dots passed through by nanotrenches. The inset is an AFM image that reveals the dark spots in this SEM image are indeed elongated particles. [file 1556-276X-6-272-S2.TIFF]

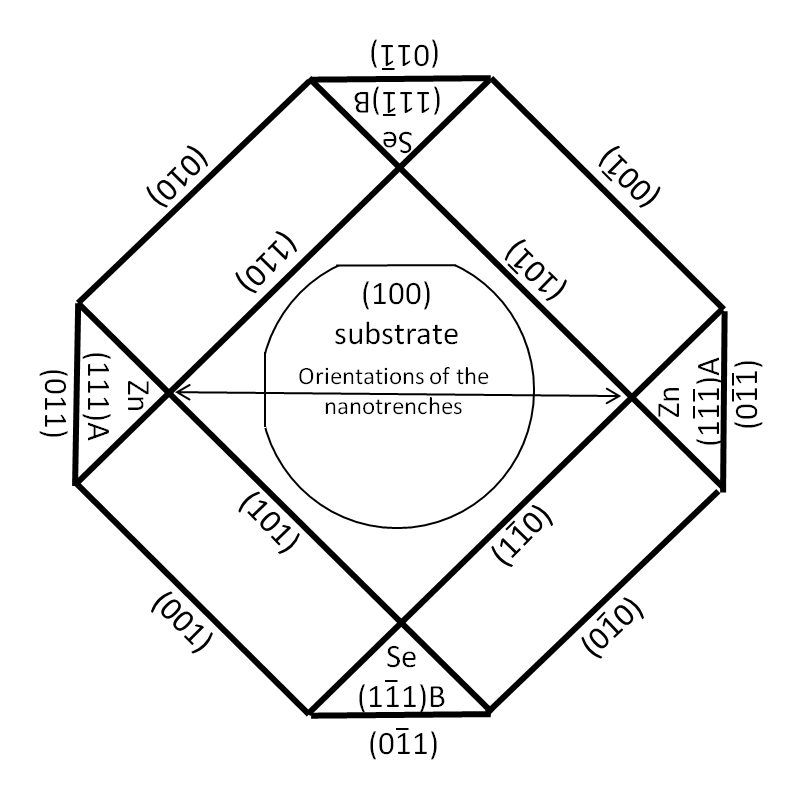

Supplement: Additional file 3 — Figure S3. Planar representation of the orientation relationship of the crystal planes of the ZnSe(100) layer. [file 1556-276X-6-272-S3.TIFF]
